# Cross-sectional study of changes in physical activity behavior during the COVID-19 pandemic among US adults

**DOI:** 10.1186/s12966-021-01161-4

**Published:** 2021-07-07

**Authors:** Kathleen B. Watson, Geoffrey P. Whitfield, George Huntzicker, John D. Omura, Emily Ussery, Tiffany J. Chen, Robyn Neblett Fanfair

**Affiliations:** 1 National Center for Chronic Disease Prevention and Health Promotion, Centers for Disease Control and Prevention, 4770 Buford Highway, NE, Mailstop, S107-5, Atlanta, GA 30341 USA; 2 National Center for HIV/AIDS, Viral Hepatitis, STD, and TB Prevention, Centers for Disease Control and Prevention, Atlanta, GA USA

**Keywords:** Health disparities, Epidemiology, Environment

## Abstract

**Background:**

Physical activity (PA) provides numerous health benefits relevant to the COVID-19 pandemic. However, concerns exist that PA levels may have decreased during the pandemic thus exacerbating health disparities. This study aims to determine changes in and locations for PA and reasons for decreased PA during the pandemic.

**Methods:**

Reported percentage of changes in and locations for PA and reasons for decreased PA were examined in 3829 US adults who completed the 2020 SummerStyles survey.

**Results:**

Overall, 30% reported less PA, and 50% reported no change or no activity during the pandemic; percentages varied across subgroups. Adults who were non-Hispanic Black (Black) or Hispanic (vs. non-Hispanic White, (White)) reported less PA. Fewer Black adults (vs. White) reported doing most PA in their neighborhood. Concern about exposure to the virus (39%) was the most common reason adults were less active.

**Conclusions:**

In June 2020, nearly one-third of US adults reported decreased PA; 20% reported increased PA. Decreased activity was higher among Black and Hispanic compared to White adults; these two groups have experienced disproportionate COVID-19 impacts. Continued efforts are needed to ensure everyone has access to supports that allow them to participate in PA while still following guidance to prevent COVID-19 transmission.

## Introduction

The World Health Organization declared the coronavirus disease 2019 (COVID-19) outbreak a pandemic on March 11, 2020 [1]. As of March 15, 2021 severe acute respiratory syndrome coronavirus 2 (SARS-CoV-2), the virus that causes COVID-19, has resulted in more than 15 million cases and 531,766 deaths in the US [2]. Adults of any age with certain underlying health conditions, including cancer, heart conditions such as coronary artery disease, obesity, severe obesity, and type 2 diabetes mellitus (T2DM), are at increased risk of severe illness from COVID-19 [3, 4]. Of particular concern, these conditions are common among US adults, with a prevalence of 42.4% for obesity [5], 28.8% for hypertension [6], 9.2% for T2DM [6], 8.4% for cancer [6], and 5.6% for coronary heart disease [6]. Moreover, some racial and ethnic minority groups are more likely to have various chronic conditions and are also being disproportionately affected by COVID-19 [7–13].

Physical activity is an important behavior that provides numerous health benefits [14, 15], many of which are particularly relevant during the COVID-19 pandemic [16]. For example, adults who are physically active have a lower risk of several chronic conditions that increase the risk of severe illness from COVID-19 including cardiovascular disease, obesity, T2DM, and some cancers [3, 4, 14]. Physical activity can also help manage some of these chronic conditions [14, 15]. Beyond these long-term benefits, physical activity also offers important acute benefits including improved sleep and decreased symptoms of anxiety [14, 15] both of which have been negatively impacted during the COVID-19 pandemic [17–21]. Additionally, emerging evidence suggests that regular physical activity may benefit immune function [22, 23] and lessen the severity of acute respiratory infections [24].

Prior to the pandemic about half of US adults met the federal aerobic physical activity guideline (guideline) and disparities existed in physical activity participation [14, 15, 25]. For example, in 2018, there was a 10 percentage point difference in the prevalence of meeting the guideline for non-Hispanic White adults (57.5%) compared to non-Hispanic Black (45.8%) and Hispanic (47.8%) adults [25]. Concerns that physical activity levels might decrease during the pandemic [26] and further exacerbate disparities may stem from a variety of factors. For example, community level prevention strategies such as stay-at-home orders and school and business closures can restrict access to places where people can be active [26]. Several US states and territories began implementing such community prevention strategies in March 2020 [27]. At various times during the pandemic common places for physical activity like parks, trails, and health clubs were closed, in addition to schools and business, in some areas. Additionally, individual-level factors such as concern about exposure to the virus [28, 29] may also influence physical activity behavior.

Because of the quickly evolving nature of the pandemic and public health response measures, it is currently unknown whether physical activity levels in the US have changed during the COVID-19 pandemic. Therefore, the study purpose, which was conducted in June of 2020, was to determine 1) self-reported changes in physical activity behavior among US adults, 2) locations where most of their physical activity was done during the pandemic, and 3) among those doing less, reasons for doing less physical activity, overall and by select characteristics. Findings can help inform efforts to promote physical activity during the COVID-19 pandemic.

## Methods

### Survey and analytic sample

Porter Novelli’s 2020 ConsumerStyles database is built from a series of web-based surveys via the Ipsos KnowledgePanel® that gathers insights about US consumers. Panel members are randomly recruited using probability-based sampling by address. The panel is continuously replenished and maintains approximately 60,000 panelists. The SpringStyles survey was fielded from 3/19/2020–4/9/2020 and was sent to a random sample of 11,097 adult panelists (aged ≥18 years) and a supplemental sample of panelists with children (response rate = 58.2%). The SummerStyles survey, fielded from 6/10/2020–6/25/2020, was sent to a random sample of 6463 SpringStyles respondents. A total of 4053 surveys were returned (response rate = 62.7%). Those who completed the survey received reward points worth approximately $5 and were entered into a monthly sweepstakes. The resulting data were weighted to match the US Current Population Survey proportions for sex, age, household income, race/ethnicity, household size, education, census region, and metro status. Institutional review board approval for this analysis was not needed; the Centers for Disease Control and Prevention was not engaged in human subjects research as personal identifiers were not included in the data file.

Data were excluded (*n* = 224; 5.5%) if they were missing information on weight status or physical activity behavior (final analytic sample = 3829). Adults with missing data were significantly more likely to be Hispanic and have a high school diploma or less compared to those with complete data.

### Measures

#### Physical activity behavior

To assess changes in physical activity behavior during the COVID-19 pandemic, respondents were asked, “During the COVID-19 pandemic, have you changed the amount of physical activity or exercise that you usually do?” Respondents were able to select one of the following options: “Yes, I did more physical activity/exercise” (more); “Yes, I did less physical activity/exercise” (less); “No, my activity level did not change” (no change); and “I do not do physical activity or exercise”. Respondents who selected more, less, or no change were then asked, “Where have you done most of your physical activity or exercise during the COVID-19 pandemic?”. Respondents were able to select all that apply from the following options: “Inside my home”, “Around my neighborhood”, “At a park or public trail”, and “Other location”. Respondents who indicated they did less activity were asked, “Why have you done less physical activity or exercise during the COVID-19 pandemic?”. They were able to select all that apply from the following options: “I worked extra hours”, “I was taking care of others (i.e., children)”, “I did not have the right equipment”, “I did not have enough space”, “I couldn’t do my preferred activity alone”, “I was concerned about virus exposure”, and “Other reason”.

To assess the status of meeting the federal aerobic physical activity guideline (guideline), modified versions of the National Health Interview Survey leisure-time physical activity questions were used [30]. Respondents were asked, during their leisure time, how often in a usual week and, if applicable, the duration they participated in (1) moderate-intensity activities (i.e., sweating or moderate increase in breathing or heart rate) and (2) vigorous-intensity activities (i.e., large increases in breathing or heart rate). Total minutes of moderate-intensity–equivalent activity was calculated by counting 1 min of vigorous-intensity activity as 2 min of moderate-intensity activity [14]. Respondents were classified as meeting (≥150 min/week of moderate-intensity–equivalent physical activity) or not meeting the guideline [14]. Based on the timing of the survey, it is assumed respondents reported usual behavior during the pandemic.

#### Demographic characteristics

Respondent characteristics included sex (male, female), age (18–44, 45–64, 65 years and older), race/ethnicity (White, non-Hispanic [White]; Black, non-Hispanic [Black]; Hispanic; non-Hispanic Other including multiracial [Other]), education level (high school diploma or less, some college, college graduate), and region (Midwest, Northeast, South, West) [31]. Weight status was calculated and classified using body mass index (weight [kg] / height [m]^2^) as: underweight or normal weight [< 25.0 kg/m^2^], overweight [25.0–29.9 kg/m^2^], obesity [≥ 30 kg/m^2^]) [32]. To assess residence, rural-urban portion [33] data was obtained through the Geocorr 2014: Geographic Correspondence Engine [34] and linked to the respondent’s zip code.

### Statistical analyses

Percentages of 1) adults who self-reported more, less, or no change in physical activity during the COVID-19 pandemic, 2) the reported location where most adult’s physical activity was done during the COVID-19 pandemic, among adults reporting more, less, or no change in physical activity, and 3) the reasons for doing less during the COVID-19 pandemic, among adults reporting less physical activity, were calculated overall and by respondent characteristics (sex, age, race/ethnicity, weight status, education, aerobic physical activity guideline, region, rural−urban residence). Chi-square tests of independence were used to determine whether each subgroup was associated with change in physical activity. Other race/ethnicity is a heterogeneous group, so their results were not interpreted. Pairwise t tests, with Bonferroni correction, and orthogonal polynomial contrasts were used to identify significant differences and trends by respondent characteristics. Two-sided *P* < 0.05 was considered significant. To help reduce survey bias and to be representative of the 2020 US population, the data were weighted based on gender, age, household income, race/ethnicity, household size, education, census region, metro status, and parental status of children 12–17 years old. Analyses were conducted in 2020 using SUDAAN version 11.0 (Research Triangle Institute, Research Triangle Park, North Carolina) to account for survey weights.

## Results

During the COVID-19 pandemic, 20.3% of US adults reported being more active, 30.4% of adults reported being less active, and 42.7% of adults reported no change in their amount of physical activity; these percentages varied significantly across subgroups (Table 1). Adults who were female (vs. male), aged 18–44 years (vs. 45 years and older), White (vs. Black), underweight/normal weight or overweight (vs. having obesity), college graduates (vs. some college or less), met (vs. did not meet) the guideline, from the Midwest (vs. South), and lived in urban (vs. rural) areas were more likely to report being more active during the pandemic. In contrast, adults who were Black or Hispanic (vs. White), had at least some college education (vs. less than some college education), and lived in urban (vs. rural) areas were more likely to report being less active during the pandemic.

**Table 1:** Prevalence of reported change in the amount of physical activity done during the COVID-19 pandemic among US adults by demographic characteristics, SummerStyles, 2020^a^

Characteristic	Sample Size		Change in physical activity (PA)			
			Prevalence			
	Unweighted	Weighted	More active	Less active	No change	Do not do PA
	N	% (SE)	% (SE)	% (SE)	% (SE)	
Total	3829	100 (−-)	20.3 (0.8)	30.4 (0.9)	42.7 (1.0)	6.6 (0.5)
Sex						
Male	1942	48.6 (1.0)	17.0 (1.1)^x^	30.2 (1.3)	47.3 (1.4)^x^	5.5 (0.7)^x^
Female	1887	51.4 (1.0)	23.4 (1.2)^y^	30.6 (1.3)	38.3 (1.3)^y^	7.6 (0.8)^y^
Age group (years)						
18–44	1267	45.1 (1.0)	24.9 (1.5)^x^	32.6 (1.6)	36.8 (1.7)	5.6 (0.9)^x^
45–64	1562	33.5 (0.9)	19.3 (1.1)^y^	29.4 (1.3)	44.9 (1.4)	6.5 (0.8)^y^
65+	1000	21.4 (0.7)	12.0 (1.1)^z^	27.3 (1.5)	51.8 (1.7)	8.9 (1.1)^z^
Race/Ethnicity						
White, non-Hispanic	2831	64.3 (1.0)	21.2 (1.0)^x^	26.5 (1.0)^x^	46.6 (1.1)^x^	5.7 (0.5)
Black, non-Hispanic	301	11.4 (0.7)	14.8 (2.2)^y^	38.0 (3.2)^y^	39.4 (3.1)^x^	7.8 (1.8)
Hispanic	386	15.8 (0.8)	17.4 (2.2)^x, y^	34.6 (2.8)^y^	38.6 (2.9)^x^	9.5 (1.8)
Other, non-Hispanic^b^	311	8.5 (0.6)	26.0 (3.2)^x^	41.9 (3.6)^y^	25.2 (3.0)^y^	7.0 (2.0)
Weight status^c^						
Underweight / normal	1261	35.6 (1.0)	23.3 (1.5)^x^	29.4 (1.6)	41.9 (1.7)	5.4 (0.9)^x^
Overweight	1306	32.3 (0.9)	21.0 (1.4)^x^	29.9 (1.6)	44.6 (1.7)	4.6 (0.7)^x^
Obesity	1262	32.0 (0.9)	16.2 (1.3)^y^	32.1 (1.6)	41.7 (1.6)	10.0 (1.1)^y^
Education level						
≤ High school diploma	1187	37.8 (1.0)	12.7 (1.2)^x^	25.2 (1.6)^x^	50.9 (1.8)^x^	11.2 (1.1)^x^
Some college	1061	27.8 (0.9)	19.8 (1.6)^y^	31.8 (1.9)^y^	43.3 (1.9)^y^	5.0 (0.8)^y^
College graduate	1581	34.4 (0.9)	28.9 (1.4)^z^	34.9 (1.4)^y^	33.3 (1.3)^z^	2.9 (0.5)^z^
Aerobic physical activity guideline^d^						
Does not meet	1707	46.2 (0.8)	12.3 (1.0)^x^	31.3 (1.4)	44.0 (1.5)	12.4 (1.0)^x^
Meets	2122	53.8 (1.0)	27.1 (1.2)^y^	29.6 (1.2)	41.6 (1.3)	1.6 (0.4)^y^
Region						
Midwest	701	17.7 (0.7)	25.2 (2.1)^x^	32.3 (2.2)	37.7 (2.2)^x^	4.8 (1.0)
Northeast	854	20.7 (0.8)	18.6 (1.6)^x, y^	31.4 (2.0)	43.5 (2.1)^x, y^	6.5 (1.1)
South	1369	38.0 (1.0)	17.8 (1.3)^y^	27.6 (1.5)	46.9 (1.6)^y^	7.7 (0.9)
West	905	23.5 (0.8)	22.0 (1.8)^x, y^	32.5 (1.9)	39.1 (2.0)^x^	6.4 (1.1)
Residence						
Rural	527	14.3 (0.7)	16.2 (2.2)^x^	20.4 (2.1)^x^	56.9 (2.8)^x^	6.4 (1.5)
Urban	3302	85.7 (0.7)	20.9 (0.9)^y^	32.1 (1.0)^y^	40.4 (1.0)^y^	6.6 (0.6)

^a^Wald Chi-square test of independence between Region and change in physical activity was *P* = 0.002; all remaining tests were *P* < 0.001

^b^Other race includes: American Indian, Alaska Native, Asian, Native Hawaiian, other Pacific Islander, and multi-racial

^c^Underweight / normal, overweight, and obesity classifications are based on the body mass index, which is weight (kg) / height (m)^2^. Underweight / normal: < 25.0; overweight: 25.0 -- 29.9; and obesity: > 30.0

^d^Participant meets the aerobic component of the physical activity guideline, per self-reported usual week, when they engage in moderate intensity ≥150 min/week, vigorous intensity ≥75 min/week, or an equivalent combination

^x, y, z^Across categories of a given characteristic, values in a column that share a letter are not significantly different from each other; values that do not have a superscript are not significantly different

**Fig. 1:**
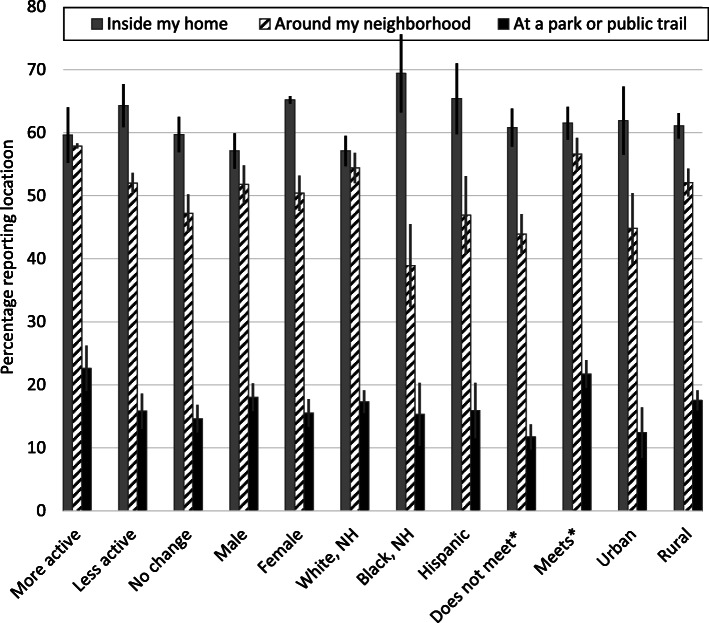
Prevalence of report locations where most physical activity was done during the COVID-19 pandemic among US adults by change in physical activity, sex, race/ethnicity, meeting guidelines, and rural—urban residence, SummerStyles 2020. ^*^Participant meets the aerobic component of the physical activity guideline, per self-reported usual week, when they engage in moderate intensity ≥150 min/week, vigorous intensity ≥75 min/week, or an equivalent combination, otherwise participant does not meet the aerobic component of the guideline. *Significant pairwise differences (all P < 0.001 unless otherwise noted):* inside my home (male vs. female; White, NH vs. Black, NH and Hispanic (*P* = 0.006)), around my neighborhood (more active vs. no change; White, NH vs. Black, NH; meets vs. does not meet guidelines; urban vs. rural), at a park or public trail (more vs. less and no change, meets vs. does not meet guidelines; urban vs. rural (*P* = 0.002))

**Fig. 2:**
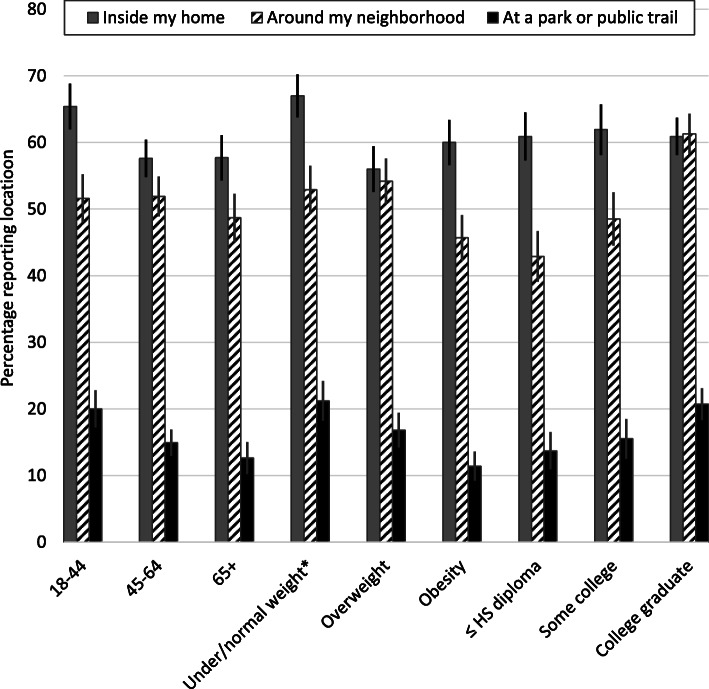
Prevalence of report locations where most physical activity was done during the COVID-19 pandemic among US adults by age, weight status, and education, SummerStyles, 2020. ^*^Underweight / normal, overweight, and obesity classifications are based on the body mass index, which is weight (kg) / height (m)^2^. Underweight / normal: < 25.0; overweight: 25.0 -- 29.9; and obesity: > 30.0. *Significant linear trends:* inside my home (age *P* = 0.002, weight status *P* = 0.003), around my neighborhood (weight status *P* = 0.004, education *P* < 0.001), at a park or public trail (age *P* < 0.001, weight status *P* < 0.001, education *P* < 0.001)

Among those who reported doing some physical activity, over half reported they did most of their activity inside their home (61.1%) and around their neighborhood (51.1%) while few reported doing most physical activity at a park or public trail (16.7%) or at another location (9.6%) (data not shown). Reported location varied significantly across most subgroups (Figs. 1, 2). For example, Black or Hispanic (vs. White) adults were more likely to report doing most of their physical activity inside the home during the pandemic whereas White (vs. Black) adults were more likely to report doing physical activity around their neighborhood; as age increased, doing physical activity at a park or public trail was less likely.

Among adults who reported less activity during the pandemic, the most common reason reported was concern about exposure to the virus (38.8%), followed by having an “other” reason (32.2%), and not having the right equipment (30.7%) (Table 2). Within reasons for less activity, there were statistically significant differences and trends by sex, age, weight status, education, usual physical activity level, and rural−urban residence. For example, among adults who were less active during the pandemic, younger adults were more likely than older adults to report their reasons being they worked extra hours, took care of others, lacked the right equipment, and had insufficient space. In contrast, older adults were more likely than younger adults to be concerned about exposure to the virus.

**Table 2:** Prevalence of reported reasons for doing less physical activity during the COVID-19 pandemic among US adults, SummerStyles, 2020^a^

Characteristics	Sample Size (unweighted)	Reasons for less physical activity (*n* = 1136)						
		Worked extra hours	Taking care of others (i.e., children)	Not have the right equipment	Not enough space	Unable to do preferred activity alone	Concerned about virus exposure	Other reason
	n	% (SE)	% (SE)	% (SE)	% (SE)	% (SE)	% (SE)	% (SE)
Total	1136	10.0 (1.1)	12.5 (1.2)	30.7 (1.7)	19.2 (1.5)	12.7 (1.2)	38.8 (1.7)	32.2 (1.7)
Sex								
Male	562	9.0 (1.3)	8.0 (1.3)^x^	36.1 (2.5)^x^	21.8 (2.2)	11.6 (1.8)	37.9 (2.5)	33.6 (2.7)
Female	574	11.0 (1.7)	16.6 (1.9)^y^	25.6 (2.3)^y^	16.7 (2.0)	13.7 (1.7)	39.6 (2.4)	31.0 (2.3)
Age group (years)								
18–44	403	12.9 (1.9)^L^	16.9 (2.1)^L^	38.0 (3.0)^L^	25.1 (2.6)^L^	14.1 (2.1)	33.9 (2.8)^L^	30.7 (3.0)
45–64	459	11.1 (1.6)	10.2 (1.7)	26.2 (2.3)	15.8 (1.9)	11.4 (1.8)	41.8 (2.6)	32.1 (2.4)
65+	274	0.9 (0.6)	5.1 (1.6)	19.7 (2.5)	9.9 (2.1)	11.2 (2.2)	45.9 (3.3)	36.2 (3.1)
Race/Ethnicity								
White, non-Hispanic	772	11.3 (1.4)	12.5 (1.4)	29.2 (2.0)	17.2 (1.7)	12.8 (1.5)	34.6 (2.0)	37.0 (2.1)
Black, non-Hispanic	107	9.4 (3.5)	11.8 (3.8)	29.7 (5.1)	22.7 (5.1)	7.2 (2.6)	41.6 (5.3)	27.0 (5.0)
Hispanic	131	8.6 (2.7)	12.3 (3.2)	32.5 (4.8)	17.0 (3.6)	10.7 (3.0)	43.4 (4.9)	29.8 (5.0)
Other, non-Hispanic^b^	126	6.8 (2.4)	13.6 (3.6)	35.8 (5.4)	27.5 (4.9)	21.5 (5.0)	48.4 (5.6)	19.5 (4.6)
Weight status^c^								
Underweight/normal	358	9.0 (1.8)	14.8 (2.3)	35.3 (3.2)^L^	22.3 (2.8)	16.6 (2.6)^L^	37.7 (3.1)	33.7 (3.1)
Overweight	372	8.5 (1.6)	10.7 (2.0)	31.1 (3.1)	17.1 (2.5)	10.9 (1.9)	37.0 (3.0)	34.0 (3.3)
Obesity	406	12.5 (2.1)	11.7 (1.8)	25.5 (2.5)	17.9 (2.4)	10.3 (1.8)	41.6 (2.9)	29.1 (2.6)
Education level								
≤ High school diploma	283	5.4 (1.8)^L^	10.5 (2.2)^L^	18.9 (2.8)^L^	15.6 (2.6)	9.8 (2.2)	39.6 (3.4)	30.8 (3.4)
Some college	310	7.6 (1.9)	9.5 (2.0)	30.4 (3.6)	19.2 (3.1)	12.4 (2.6)	42.1 (3.6)	34.4 (3.7)
College graduate	543	15.4 (1.8)	16.3 (1.9)	40.1 (2.4)	22.0 (2.2)	15.1 (1.8)	35.6 (2.3)	31.8 (2.2)
Aerobic physical activity guideline^d^								
Did not meet	530	9.0 (1.5)	12.5 (1.8)	20.4 (2.2)^x^	15.5 (1.9)^x^	13.2 (1.9)	42.1 (2.6)	35.3 (2.6)
Met guideline	606	10.9 (1.6)	12.4 (1.6)	40.0 (2.5)^y^	22.5 (2.2)^y^	12.2 (1.6)	35.7 (2.3)	29.4 (2.3)
Region								
Midwest	226	8.3 (2.1)	11.9 (2.6)	31.1 (4.0)	21.1 (3.5)	11.7 (2.8)	36.1 (3.7)	37.7 (4.0)
Northeast	254	12.1 (2.5)	10.9 (2.3)	27.5 (3.5)	16.9 (3.1)	11.5 (2.2)	34.4 (3.6)	36.0 (3.8)
South	360	12.3 (2.2)	13.4 (2.2)	28.0 (2.7)	17.3 (2.6)	11.7 (2.2)	39.7 (3.1)	30.0 (3.0)
West	296	6.3 (1.6)	13.0 (2.4)	36.6 (3.6)	22.3 (3.0)	15.7 (2.7)	43.3 (3.5)	28.2 (3.3)
Residence								
Rural	119	11.3 (3.1)	12.2 (3.7)	16.9 (3.8)^x^	11.4 (3.1)^x^	7.1 (2.5)^x^	43.4 (5.5)	42.0 (5.8)
Urban	1017	9.9 (1.1)	12.5 (1.3)	32.1 (1.8)^y^	20.0 (1.6)^y^	13.2 (1.4)^y^	38.3 (1.8)	31.2 (1.8)

^a^Significant pairwise differences and trends (all *P* < 0.001 unless otherwise noted): taking care of others (education trend *P* = 0.05), not the right equipment (male vs. female *P* = 0.002; weight status trend *P* = 0.02; rural vs. urban *P* = 0.01), not enough space (meet vs. not meet guideline *P* = 0.02; rural vs. urban *P* = 0.01), unable to do preferred activity alone (weight status trend *P* = 0.05; rural vs. urban *P* = 0.03), concern about virus (age trend *P* = 0.005)

^b^Other race includes: American Indian, Alaska Native, Asian, Native Hawaiian, other Pacific Islander, and multi-racial

^c^Underweight / normal, overweight, and obesity classifications are based on the body mass index, which is weight (kg) / height (m)^2^. Underweight / normal: < 25.0; overweight: 25.0 -- 29.9; and obesity: > 30.0

^d^Participant meets the aerobic component of the physical activity guideline, per self-reported usual week, when they engage in moderate intensity ≥150 min/week, vigorous intensity ≥75 min/week, or an equivalent combination

^x, y^Within categories of a given characteristic, values in a column that share a letter are not significantly different from each other

^L^For ordinal variables, linear trends across the categories were assessed; Superscript (L) indicates a positive or negative linear trend

## Discussion

In June 2020, 20% of US adults reported doing more physical activity during the COVID-19 pandemic while 30% reported doing less. Reporting less physical activity was more common among adults who were Black and Hispanic, had at least some college education, and lived in urban areas compared to their counterparts. Fewer Black (vs. White) adults and adults with obesity (vs. lower weight categories) reported getting most of their physical activity in their neighborhood. The most frequently reported reason for doing less physical activity was concern about exposure to the virus (39%). Understanding changes in physical activity behavior during the COVID-19 pandemic and underlying reasons for less activity can help identify groups that may benefit from efforts to increase physical activity, thus, maximizing the public health benefits from this important health behavior.

We observed that groups that have been found in prior research to experience severe burdens from COVID-19 morbidity and mortality (i.e., Black and Hispanic populations) [35] were also more likely to report doing less physical activity. Additionally, fewer Blacks than Hispanics or Whites reported being active in their neighborhood. Long-standing systemic health and social inequities have led to communities of predominantly minority racial and ethnic groups and lower-income residents lacking features supporting walking, having limited access to safe, well-maintained parks, and experiencing higher crime rates [36]. Lack of access to supports and opportunities to safely engage in physical activity, combined with disproportionate burdens of COVID-19 and chronic disease during the COVID-19 pandemic, has highlighted the importance of implementing community-based strategies [37] to increase physical activity, particularly for racial and ethnic minority groups.

Our study examines self-reported changes in physical activity during the pandemic by demographic characteristics which few other studies to date have done. Knell et al. surveyed adults between April 15–May 5, 2020 and observed, consistent with our study, more adults reported decreased (39%) versus increased (25%) physical activity [38]. Although women were more likely to report an increase than men, no other significant differences in change were reported by age, race/ethnicity, education, or weight status [38]. Potential explanations for the varying findings by subgroup from our study may be that Knell et al. [38] surveyed adults primarily from Texas whereas our study was nationwide and weighted to the US population, and our study was conducted slightly later in the pandemic [2].

Beyond the US, studies from other countries have also examined changes in physical activity during the COVID-19 pandemic. Among young German adults, a substantial percentage point difference was observed between those who reported being less (45%) and more (33%) active during the pandemic after implementation of a government lockdown [39]. We observed similar patterns but with varying magnitudes, though we were unable to link changes to prevention efforts impacting the respondent at the time of the survey. In Canada, similar proportions of adults reported being more (36%) and less (35%) active after COVID-19 restrictions were put in place [40]. When stratified by activity level, 41% of inactive (defined as not meeting the guideline of 150 min of moderate-intensity equivalent minutes per week) Canadian individuals became less active while 33% became more active; in contrast, we observed that 31% of adults not meeting the guideline reported being less active and 12% reported being more active. Among active Canadian adults, 22% became less active and 40% became more active [40], which were similar to our percentages reporting less (30%) and more (27%) activity. Among adults participating in insufficient levels of physical activity to meet the guidelines, reasons for differences in being more or less active between studies are unclear. Future work examining factors driving behavior change among insufficiently active adults may assist in the planning and implementation of strategies to help this population obtain sufficient levels of physical activity for substantial health benefits.

Although examining the characteristics of adults who reported being less active during COVID-19 pandemic, examining the characteristics of adults who were more active is also important. Adults who were female, younger (aged 18–44 years), White, not having obesity, a college graduate, lived in urban areas, and from the Midwest reported being more active during the COVID-19 pandemic. Understanding the subgroups which were more active may also help inform population-level strategies that could be transferred to other groups or in times of non-pandemic.

While community prevention strategies were necessary to slow disease transmission and save lives, some research has linked these strategies to reduced physical activity. Some studies related to the COVID-19 pandemic have assessed changes in physical activity following implementation of community prevention strategies using activity tracker data. Across several countries, studies observed that decreases in steps [41–43] or minutes of moderate-to-vigorous intensity activity per week [44, 45] were related to lockdown policies, particularly more restrictive policies [41–45]. Although we were unable to link individual data to community prevention strategies [27], our self-reported findings of adults being less active during the pandemic in June 2020, a period when some prevention strategies were in place across the nation [27], are consistent with findings from other studies [41–45]. While such studies are useful in examining the impact of prevention strategies on physical activity, few have included data on individual characteristics to examine impacts on specific subpopulations. Future work linking community prevention strategies, physical activity, and subgroup characteristics may help identify areas where the prevention strategies, such as stay-at-home orders, may possibly impact physical activity and worsen disparities [26].

In terms of locations where adults participated in physical activity during the COVID-19 pandemic, our study, along with others conducted in Canada and the US, observed that most adults report being physically active at home, including the garage, or in their neighborhood; fewer adults report doing so at a park or trail [40, 41]. Moreover, we observed that adults with higher educational attainment are more likely to be active in their neighborhood compared to their counterparts, similar to findings from other studies using income as a measure of socioeconomic status [41]. Although reasons for these differences in location are unknown, a number of reasons related to location may influence physical activity behavior, e.g. presence and/or safety of places, proximity of these places, weather, other obligations keeping persons at home, or personal preference. Understanding why people chose different places can provide information to practitioners and professionals about how to keep people active during the pandemic [46–48]. For example, professionals in some communities have introduced “safe, slow streets” that provide places to recreate safely on city streets [49], while some have worked to address changes in travel demand and the need for social distancing in other ways such as free bike share programs [50].

Few studies have examined reasons individuals have been less active during the pandemic, and ours is unique in examining such reasons by subgroup populations. One study observed that the most common reason was worry or stress about health, finances, and job security, followed by resource concerns, including limited locations to engage in physical activity [38]. The most common reason we observed overall and among most subgroups (highest in adults ≥65 years) was concern about exposure to the virus, which was also the only health concern we assessed. The second most common reason we observed (apart from an “other” reason) was not having the right equipment. Additional studies, including qualitative analyses examining barriers to physical activity during the pandemic, may help identify whether effective strategies to increase physical activity developed prior to the onset of the COVID-19 pandemic [14, 37, 51, 52] might benefit from modification in pandemic settings.

This study is subject to limitations. First, use of an internet panel survey of volunteers may introduce sample selection bias. However, previous research found general equivalence between random-digit dialing and panel approaches [53, 54]. Second, survey data are self-reported and the validity and reliability of survey questions about physical activity during the pandemic are unknown. Third, we were unable to account for prevention strategies or indicators of community transmission severity (e.g., COVID-19 infection rates, hospitalizations, deaths) where respondents lived which may have influenced physical activity behaviors. Fourth, we were unable to ascertain disability status which may limit or affect their physical activity. Despite these limitations, strengths include the timeliness of data and use of a diverse, nationwide sample allowing stratification by various characteristics. Fifth, our study was limited in what we were not able to measure. We were unable to separately assess changes in key domains of physical activity, especially leisure and transportation, or to assess the main location for physical activity prior to the pandemic. The lists for physical activity locations and reasons for less physical activity were not comprehensive. However, the study still provides information that it useful to practitioners and professionals. Finally, we may have an incomplete picture of physical activity across the nation because some parts of the country had not experienced outbreaks by June 2020. It is possible, even likely, that physical activity habits were much different during the peak periods of late summer 2020 and winter 2021.

## Conclusion

Regular physical activity provides numerous health benefits, many of which are particularly relevant during the COVID-19 pandemic. Even though there were some increases in physical activity, our findings suggest physical activity levels have decreased among almost one-third of US adults. Decreased activity was higher among Black and Hispanic (vs. White) adults who are also known to be at risk for severe illness due to COVID-19 and have higher rates of chronic diseases. Continued efforts are needed to ensure everyone has access to supports that allow them to participate in physical activity while still following guidance to prevent COVID-19 transmission. Some population subgroups disproportionately impacted by COVID-19 may benefit from more focused efforts to help mitigate barriers to being physically active during the pandemic.

## Data Availability

This study analyses data owned by Porter Novelli Public Services Inc., a third party, so we are unable to archive or share the raw data.
